# Intestinal region-specific Wnt signalling profiles reveal interrelation between cell identity and oncogenic pathway activity in cancer development

**DOI:** 10.1186/s12935-020-01661-6

**Published:** 2020-12-03

**Authors:** Ronja S. Adam, Sanne M. van Neerven, Cayetano Pleguezuelos-Manzano, Salvatore Simmini, Nicolas Léveillé, Nina E. de Groot, Andrew N. Holding, Florian Markowetz, Louis Vermeulen

**Affiliations:** 1 Laboratory for Experimental Oncology and Radiobiology (LEXOR), Center for Experimental and Molecular Medicine (CEMM), Cancer Center Amsterdam and Amsterdam Gastroenterology and Metabolism, Amsterdam University Medical Centers, Meibergdreef 9, 1105 AZ Amsterdam, The Netherlands; 2 Oncode Institute, Meibergdreef 9, 1105 AZ Amsterdam, The Netherlands; 3 Hubrecht Institute, Royal Netherlands Academy of Arts and Sciences (KNAW) and UMC Utrecht, Uppsalalaan 8, Utrecht, 3584 CT The Netherlands; 4 Research & Development Department at STEMCELL Technologies UK, 7100 Cambridge Research Park, Beach Drive Waterbeach, Cambridge, CB25 9TL UK; 5 Cancer Research UK Cambridge Institute, University of Cambridge, Robinson Way, Cambridge, CB2 0RE UK; 6 The Alan Turing Institute, 96 Euston Road, Kings Cross, London, NW1 2DB UK; 7 University of York, Wentworth Way, York, YO10 5DD UK

**Keywords:** Intestinal cancer, Wnt signalling, Cell of origin

## Abstract

**Background:**

Cancer results from the accumulation of mutations leading to the acquisition of cancer promoting characteristics such as increased proliferation and resistance to cell death. In colorectal cancer, an early mutation leading to such features usually occurs in the *APC* or *CTNNB1* genes, thereby activating Wnt signalling. However, substantial phenotypic differences between cancers originating within the same organ, such as molecular subtypes, are not fully reflected by differences in mutations. Indeed, the phenotype seems to result from a complex interplay between the cell-intrinsic features and the acquired mutations, which is difficult to disentangle when established tumours are studied.

**Methods:**

We use a 3D in vitro organoid model to study the early phase of colorectal cancer development. From three different murine intestinal locations we grow organoids. These are transformed to resemble adenomas after Wnt activation through lentiviral transduction with a stable form of β-Catenin. The gene expression before and after Wnt activation is compared within each intestinal origin and across the three locations using RNA sequencing. To validate and generalize our findings, we use gene expression data from patients.

**Results:**

In reaction to Wnt activation we observe downregulation of location specific genes and differentiation markers. A similar effect is seen in patient data, where genes with significant differential expression between the normal left and right colon are downregulated in the cancer samples. Furthermore, the signature of Wnt target genes differs between the three intestinal locations in the organoids. The location specific Wnt signatures are dominated by genes which have been lowly expressed in the tissue of origin, and are the targets of transcription factors that are activated following enhanced Wnt signalling.

**Conclusion:**

We observed that the region-specific cell identity has a substantial effect on the reaction to Wnt activation in a simple intestinal adenoma model. These findings provide a way forward in resolving the distinct biology between left- and right-sided human colon cancers with potential clinical relevance.

## Background

Activation of Wnt signalling is seen in a variety of malignancies [1, 2], and is particularly crucial in colon cancer [3]. Besides its role in cancer, Wnt signalling is tightly regulated in embryonal development and tissue homeostasis [4–6]. Activation of the Wnt pathway leads to an increase in proliferation rate and is sufficient to initiate intestinal adenoma formation [7]. Mutations in key components of the Wnt pathway are found in > 90% of colon cancer [8]. In wild type cells, Wnt family protein members (e.g. Wnt-2b, Wnt-3) serve as ligands to activate Wnt signalling [9]. Eventually the transcription cofactor β-Catenin (encoded by *CTNNB1*) promotes Wnt target gene expression via the transcription factor family TCF [10, 11]. Principal target genes include for example the oncogene c-Myc and the cell cycle regulator Cyclin D1 [8, 12]. In absence of Wnt ligands, the β-Catenin destruction complex, comprising of APC, Axin and GSK3β, phosphorylates β-Catenin to initiate ubiquitin-dependent degradation [13, 14]. Stabilization of β-Catenin can be achieved directly by mutation of phosphorylation sites in β-Catenin [15] or indirectly by inactivation of the destruction complex (mostly through *APC* inactivating mutations). Both mechanisms are found to increase Wnt signalling in early carcinogenesis in the intestine.

It has been shown that the mutational profile is not sufficiently explaining heterogeneity between tumours [16]. It is speculated that the cell of origin, i.e. the normal cell that acquires the first cancer-promoting mutation, might have a profound impact as well [17]. For example, in hematopoietic stem cells it was even shown that the same mutation can give rise to different phenotypes, creating two subsets of stem cells with opposing behaviour resulting from the same genetic event [18]. In medulloblastoma, two molecular subtypes caused by different oncogenic pathways could be explained by distinct cells of origin [19]. However, it remains largely undefined to what extent the cell of origin determines the resulting tumour in the intestine. We propose that the longitudinal or craniocaudal axis in the intestine is an attractive model to study how the characteristics of cancer initiating cells influence the transformation process, using the confined variation of regional differentiation in the same organ. Indeed, organoid cultures established from different regions of the murine intestine were shown to conserve a location specific phenotype [20]. In addition, important earlier work form Leedham et al. revealed that there exists a gradient of Wnt activity along the longitudinal axis in both mice and humans, translating into distinct stem cell dynamics as distinct Wnt activating mutations along the intestinal tract [21]. However, how exactly the cell of origin and the acquired mutations shape the gene expression profile and phenotype of the resulting pre-malignant clones remains elusive. Dissecting the interplay of the cancer initiating cells and oncogenic mutations in the gut is of potential great relevance as in colon cancer the location of the tumour has direct clinical implications.

In colon cancer the origin in the left or the right colon has an impact on the prognosis, right-sided being associated with a worse prognosis [22]. Right-sided tumours tend to be more advanced, larger and more frequently poorly differentiated [23, 24]. This is not only a result of more advanced stage at presentation of disease but the molecular subtypes and underlying pathophysiological mechanisms also differ between the locations [16]. To identify the role of intestinal location, earlier studies looked at differential expression along the intestinal craniocaudal axis. A gene expression comparison between left and right-sided colon cancer found *PRAC1*, *HOX6C* and *HOX13B* as most differentially expressed [25]. This raises the question to which extend regional differences in colon cancer can be the result of pre-existing differences in the origin tissue. A study that did compare normal and tumour samples from caecum and sigmoid/rectosigmoid found that most genes regulated in tumours did not overlap with genes significantly different between the locations [26]. However, microsatellite status was available for less than half of the tumours, thus a difference in the molecular pathophysiology between the locations could not be excluded. To study the effect of tumour location with the same underlying pathomechanism, in this case early Wnt activation by means of *Apc* inactivation, Reichling et al. compared early adenomas from *Apc*^Min/+^ mice between small or large intestinal locations and to matched normal tissue [27]. Only a limited number of genes and no known Wnt target genes were found differentially expressed in both locations. Further studies of adenoma formation in mice observed a caudal shift of the tumour location from small intestine towards colon after introducing an additional knockout of *Smad3* in the *Apc*^Min/+^*Smad3*^−/−^ mouse or a heterozygous mutation of *Cdx2* in the *Apc*^+/Δ716^*Cdx2*^+/−^ mouse [28, 29]. These studies confirm that the cell of origin in concert with specific mutations shape the ability of the intestine to transform as well as the properties of the resulting neoplasm.

In this study we set out to further unravel the interaction of the cell of origin and the effect of an oncogenic mutation activating the Wnt pathway at three different intestinal locations. We took advantage of location specific organoid cultures to limit the influence of the microenvironment and solely focus on the direct interplay of the cell of origin and oncogenic pathway activation. Since this model is largely based on regional differences in transcriptional profiles and does not reach single cell resolution to study cell state differentiation markers, our findings reflect intestinal region-specific cellular features. We found that Wnt pathway activation partially obliterates the location specific gene expression pattern, but at the same time installs novel region-of-origin-specific transcriptomic features that cannot be inferred from the non-transformed counterparts. Human gene expression data confirmed these findings stressing the relevance of these insights also for colon cancer. This study highlights the complex interplay of regional cell type-specific features and oncogenic pathway activation in the intestine, and it establishes important context specific effects of Wnt signalling during tumour initiation.

## Results

### Organoid model of intestinal transformation

**Fig. 1:**
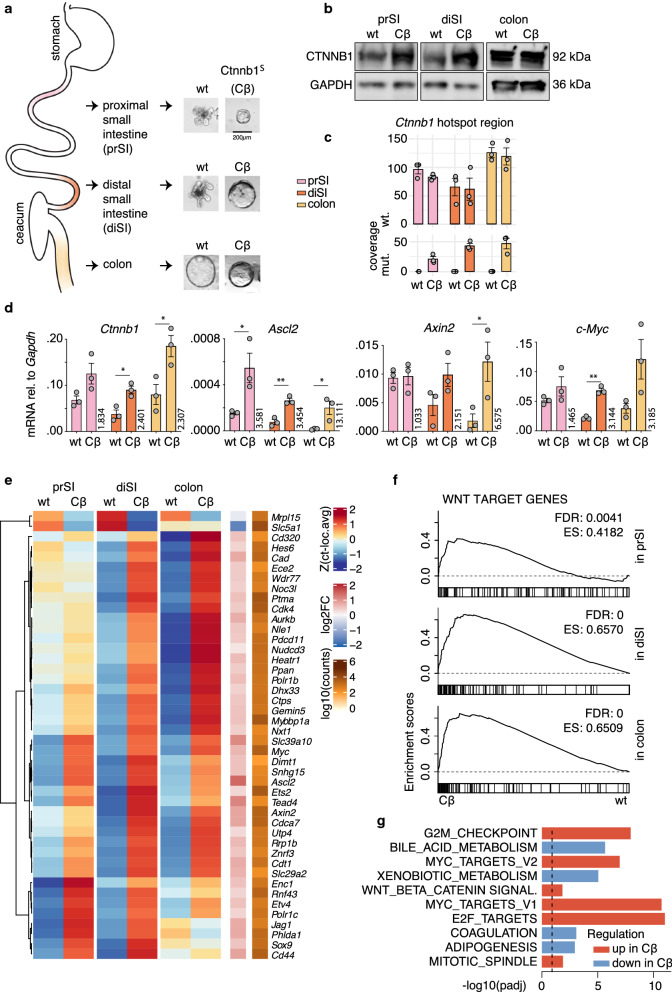
Model for Wnt activation. **a** Organoids were grown from 3 locations of the wild type (wt) murine intestine, and after transduction with stable *Ctnnb1*^*S*^ (Cβ) a cystic morphology can be observed in all locations. **b** Immunoblot of CTNNB1 and loading control GAPDH in organoids grown without (wt) or with transduced Ctnnb1^S^ (Cβ). **c** Number of reads matching the wild type (wt.) hotspot region of *Ctnnb1* and the mutated (mut.) hotspot region of *Ctnnb1*:*c.97T* > *G;109T* > *G;121A* > *G;133T* > *G*. **d** Quantitative PCR of *Ctnnb1* and important Wnt target genes, n = 3, *p < 0.05, **p < 0.005 (Student’s t-test), mean fold change in vertical number. All error bars represent S.E.M. **e** Expression of Wnt target genes [32] as average (n = 3) scaled count, centred around the location average. Right columns show summarized average logarithmic fold change (log2FC) of Cβ/wt and mean read count across locations. Indicated gene symbols are significantly differentially expressed in the average of all locations (adjusted p-value < 0.05, Wald -Test). **f** Gene Set Enrichment Analysis of Wnt target genes [32] in each location separately using GSEA preranked mode or (**g**) testing enrichment of Hallmark gene sets [50] across all locations using EGSEA

In order to study the interplay between the transcriptional profiles of cancer initiating cells and oncogenic pathway activation, we employed an in vitro model for intestinal transformation (Fig. 1a). Intestinal epithelial organoid cultures were established from three distinct regions of the murine intestine: proximal small intestine (prSI), distal small intestine (diSI), and colon. Subsequently, the organoids were transduced with an oncogenic variant of *Ctnnb1* (mutated to p.S33A;S37A;T41A;S45A, hereafter referred to as *Ctnnb1*^*S*^). The resulting stabilized β-Catenin protein is not effectively targeted for degradation, accumulates and translocates to the nucleus in order to interact with TCF family members and drive expression of Wnt target genes [15]. Following overexpression of *Ctnnb1*^*S*^, prSI and diSI organoids display a cystic growth pattern, reminiscent of organoids in which the negative Wnt regulator *Apc* has been inactivated (Fig. 1a, Additional file 1A) [30, 31]. Colonic organoids always show this cystic growth pattern [32]. To validate that CTNNB1 protein was present at increased but physiological levels after transduction, we compared the protein levels in all locations (Fig. 1b). We generated global transcriptomic RNA sequencing data from wild type (wt) organoids and *Ctnnb1*^*S*^ transduced organoids from all three locations. We verified based on the read counts of the mutation hotspot region that wild type *Ctnnb1* was similarly expressed in wt and *Ctnnb1*^*S*^ organoids, and the mutated *Ctnnb1*:*c.97T* > *G;109T* > *G;121A* > *G;133T* > *G* was only detectable following *Ctnnb1*^*S*^ transduction (Fig. 1c). Using quantitative PCR, we confirmed increased levels of *Ctnnb1*^*S*^ expression in the models as well as increased expression of the Wnt target genes *Ascl2*, *Axin2* and *c-Myc* (Fig. 1d). Next, we evaluated the expression of genes previously identified as Wnt target genes relevant in colon cancer [32] in the RNA sequencing data (Fig. 1e, f). This analysis revealed a general overexpression of Wnt target genes following *Ctnnb1*^*S*^ expression as expected, although regional differences were detected. Moreover, we employed a gene set enrichment analysis (GSEA) to identify gene expression profiles that are significantly altered following Wnt signalling activation compared to the wt organoids (Fig. 1g). This established that *c-Myc* target gene signatures and gene signatures relating to the cell cycle and proliferation were more abundantly expressed just like the Wnt signalling gene signature itself. In combination, our data reveal that this in vitro model system exhibits features reminiscent of intestinal transformation.

### Oncogenic Wnt activation erases region of origin related cell identity

**Fig. 2:**
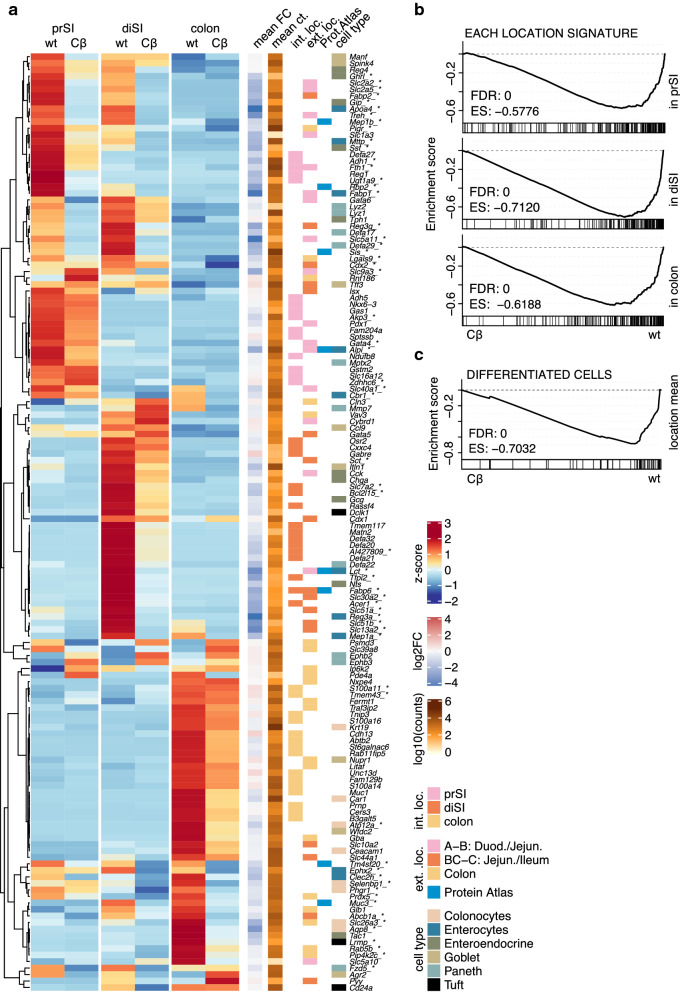
Location signatures are downregulated upon Wnt activation. **a** Heatmap shows average (n = 3) scaled counts for both conditions of each location, additional columns show summarized mean log2FC(Cβ/wt) and mean read count across locations. Genes marked with asterix are significantly differentially expressed in the average of all locations (adjusted p-value < 0.05, Wald-Test). Internal location (int. loc.) marker genes are genes which showed the most significant (Wald-Test) upregulation in the wt samples of prSI, diSI or colon compared to all other locations’ wt samples, respectively. External location (ext. loc.) marker genes were previously reported to be specific to intestinal divisions [20, 33] or specifically enriched in the intestine based on the Human Protein Atlas [34–37]. Cell type marker genes were previously reported to be specific to differentiated intestinal cells [38–40]. **b** Our own location signature exhibited a significant downregulation upon Wnt activation in each location separately using Gene Set Enrichment Analysis GSEA preranked mode. **c** Cell type marker genes [38–40] also showed significant downregulation upon Wnt activation in Gene Set Enrichment Analysis using GSEA preranked mode

To investigate how active Wnt signalling modifies region-specific gene expression profiles in intestinal epithelial cells, we identified internal sets of location marker genes. We plotted 15 genes (Fig. 2a) or 200 genes (Additional file 1B) that are significantly higher expressed in wt organoids of each location as compared to the other intestinal regions. Almost uniformly, these genes were downregulated following Wnt pathway activation, indicating that oncogenic Wnt pathway activation erases part of the location specific cell identity (Additional file 2 lists fold changes). This was also confirmed by GSEA for gene sets encompassing the 200 most significant location specific genes (Fig. 2b), demonstrating for all locations a downregulation of gene sets that represent location defined cell identity. We detected the same trend when we used external location signatures which were previously reported (Fig. 2a) [20, 33], or when we employed an in vivo derived set of genes which were found to be specifically enriched in the intestine by the Human Protein Atlas (Fig. 2a) [34–37]. Since Wnt signalling is known to be an important driver of differentiation during embryogenesis, but maintains stemness in adult intestines, we were curious how our findings relate to the level of cell type specificity. We observed that genes marking differentiated cells, as described for specific cell types in single cell gene expression studies [38–40], were significantly downregulated in *Ctnnb1*^*S*^ expressing organoids (Fig. 2a, c). From each region we selected two location specific genes based on the highest significance and fold change to validate with quantitative PCR that the expression decreased after Wnt activation (Additional file 1C). We confirmed the location specificity and loss after transformation on protein level for the growth factor Regenerating islet-derived 1, REG1, the ileal Fatty acid binding protein 6, FABP6*,* and the mucin protein Mucin-1, MUC1 (Additional file 1D). Taken together, these analyses indicate that oncogenic Wnt activation reduces expression of genes that are characteristic for the specific cellular identity associated with the intestinal location.

**Fig. 3:**
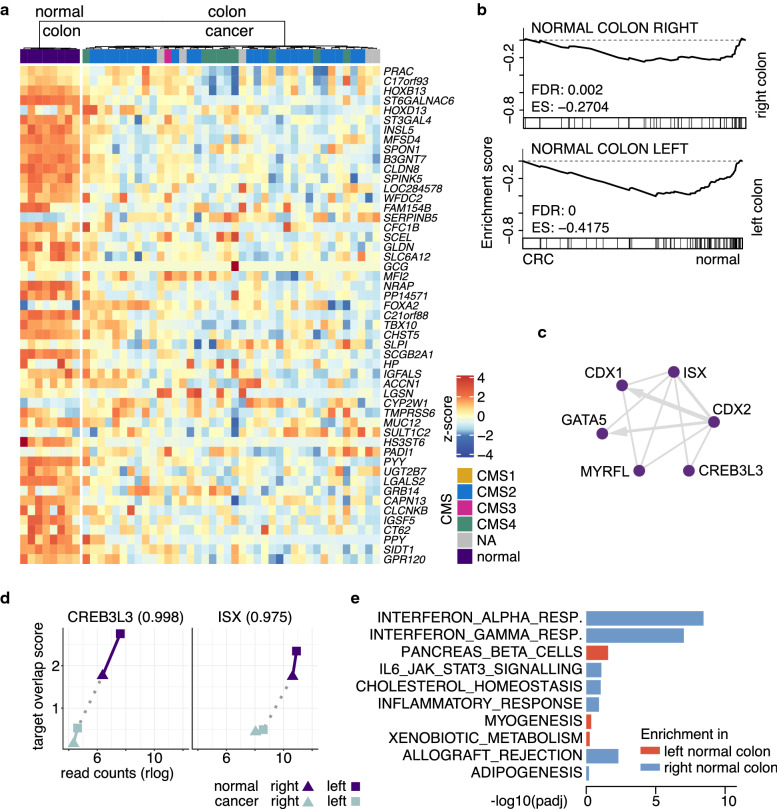
Location specific signature partially erased in human colon cancer. **a** With transcriptome data from TCGA [8] we identified genes with the most significant upregulation in left colon versus right colon (log2FC > 1, adjusted p-values < 0.01, Wald-Test) in the normal tissue samples (left panel). The expression of those genes in MSS *APC* mutated colon cancer samples of the left colon is shown in the right panel. **b** The colon side-specific signatures were analysed individually using GSEA. **c** Genes differentially expressed between left and right TCGA normal colon tissue samples were tested for overlap with transcription factor target gene sets using ChEA3 mean ranks with interaction [79]. Resulting ChEA3 scores were compared to their expression in normal and colon cancer samples and in CREB3L3 and ISX we found a high Pearson-correlation, indicated in brackets (**d**). Genes differentially expressed between left and right TCGA normal colon tissue samples were tested for enrichment in Hallmark genes sets [50] using EGSEA (**e**)

In order to evaluate if this phenomenon can also be captured in human cancers we established location specific profiles using the The Cancer Genome Atlas (TCGA) dataset. Because small intestinal cancer is very rare and left versus right colon cancers represent clinically relevant distinct entities we focus on this distinction. We selected samples from the normal colon and defined genes with the most significant differential expression between the right colon (caecum and ascending colon) and the left colon (descending colon and sigmoid). Interestingly, among the significant side-specific genes from the human normal colon, we found a significant correlation with the location specific genes from our comparison of murine colon vs. small intestine organoids, suggesting that our model reflects a general proximal vs. distal effect (Additional file 1E). Next, we selected colon adenocarcinoma samples that harbour Wnt pathway activating mutations in the *APC* gene. We included only cancer samples from microsatellite stable (MSS) cancers, since microsatellite instable cancers are a clearly distinct entity characterized by distinct pathogenesis [41–43]. The most important clinical features are summarized in Additional file 3A. In line with our in vitro findings using murine organoids, also in these patients’ cancer samples, the location specific gene expression profile is significantly impaired confirming that Wnt pathway activation obliterates location specific gene expression patterns (Fig. 3a, b, Additional file 3B). In left-sided colon cancers, the consensus molecular subtype (CMS) 2 is overrepresented and thus the Myc pathway is more activated than in right-sided colon cancers (Additional file 4B). We previously established that activation of the Wnt pathway is more prominent in CMS2 than in other CMSs [16]. This might explain why the downregulation of location markers is more pronounced in left-sided cancer samples. To identify transcription factors that could be involved in the regulation of the genes differentially expressed between left and right colon, we tested which transcription factor target gene sets were overlapping. We identified the target gene sets of CDX2, CDX1, ISX, CREB3L3 and GATA5, all reported as linked to differentiation in intestinal cells with differences along the intestinal axis [44–48] (Fig. 3c). Of note, the genes encoding these transcription factors were themselves not significantly differentially expressed between left and right normal colon (Additional file 3C), indicating another activation mechanism. However, in a subset we found the transcription factor expression downregulated in colon cancer, correlating with a decreasing overlap of their targets (Fig. 3d). To test which genes were building the location marker sets in normal tissue in vivo, we performed a GSEA on 50 curated Hallmark gene sets from the Molecular Signature Database [49–51] and found that gene sets associated with inflammatory response tended to be higher in the right colon (Fig. 3e). It is interesting to note that immune activated microsatellite instable tumours, which we excluded from our analysis, are known to be predominantly located in the right colon. The fact that inflammatory response pathways (interferon α, interferon γ, allograft rejection) were already significantly enriched in the right normal colon, suggests an inherent location specific mechanism influencing the immune microenvironment.

### Oncogenic Wnt activation uncovers a region of origin specific expression pattern

**Fig. 4:**
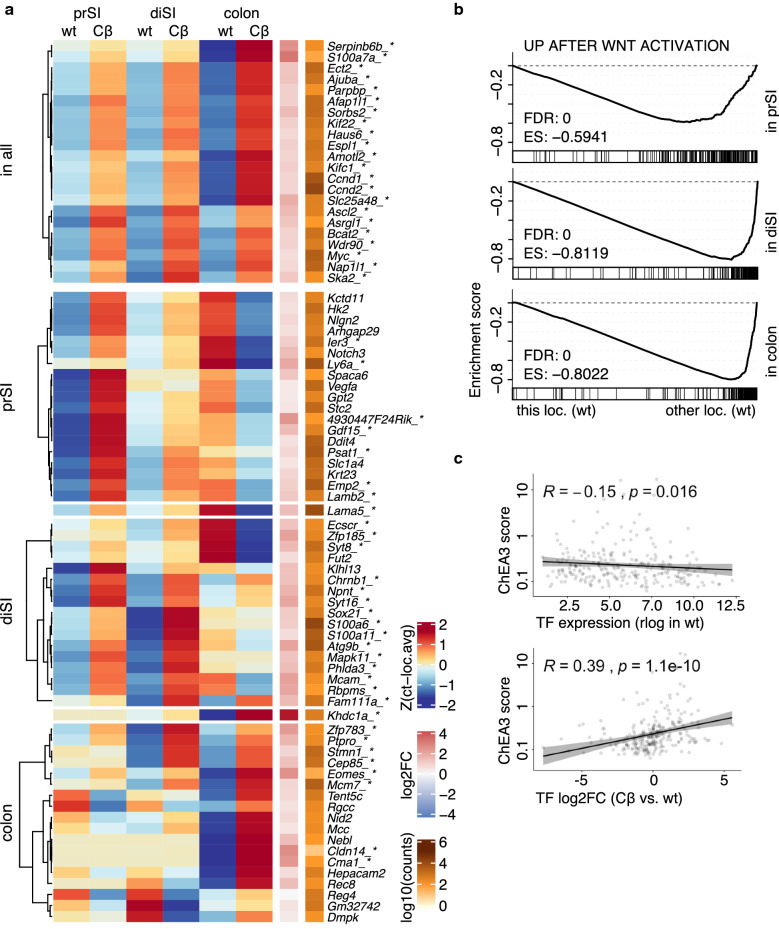
Wnt response is partly location specific. **a** Genes upregulated after Wnt activation (fold change > 1.2, adjusted p-value < 0.05, Wald-Test) were identified in each region separately. Genes which appeared significantly upregulated in all locations individually are shown on top, followed by remaining 20 most significantly upregulated genes for each location (intersecting genes shown in between). Heatmap shows average (n = 3) scaled counts per condition, centred around the location average, right columns show summarized log2FC(Cβ/wt) and mean read count across locations. Genes marked with asterix are differentially expressed in the average of all locations (adjusted p-value < 0.05, Wald-Test). **b** Gene Set Enrichment Analysis of genes upregulated after Wnt activation (fold change > 2, 200 lowest adjusted p-values < 0.05, Wald-Test) in each location using GSEA preranked mode, after genes were ranked based on expression in each location’s expression in wt vs. other locations expression in wt. **c** The genes upregulated after Wnt activation from each location were examined for overlap with transcription factor target genes using ChEA3, the resulting score was tested for correlation with the expression of the corresponding transcription factor coding gene in each location

Importantly, in addition to maintaining expression of genes characteristic of cell identity during transformation, the region of origin can also impact on the transcriptomic profiles of cancers in a different fashion. It is perceivable that activation of the Wnt pathway results in activation of genes in a location specific fashion, thereby installing a novel location specific identity. To test this hypothesis, we identified the Wnt target genes for each location separately, by comparing the expression profiles of the wt organoids of the different regions with the respective *Ctnnb1*^*S*^ counterparts (Fig. 4a). This analysis revealed that following Wnt activation the organoids from different regions respond by activating partially distinct sets of Wnt target genes. For a substantial set of genes even an inverse relationship was detected, i.e. activation upon Wnt signalling in one location and downregulation in *Ctnnb1*^*S*^ organoids from another location. This suggests that Wnt activation installs an otherwise undetected location specific gene expression program. We were interested in detailing the nature of the genes that were activated in a region of origin specific fashion. With GSEA we found that the upregulated Wnt target genes tended to be lowly expressed before Wnt activation (Fig. 4b). To test statistically, which genes responded to Wnt activation differently between the locations, we applied a likelihood ratio test for the effect of the Wnt activation depending on the location. The resulting genes were assigned to the three locations based on where they showed the maximum (positive) log2 fold change (Additional file 4A). To identify transcription factors which could explain the regional differences, the upregulated Wnt target genes of each location were tested for overlap with transcription factor target gene sets. We found that the expression levels of the transcription factors with high evidence for overlapping target genes were rather low in wild type and showed upregulation upon Wnt activation (Fig. 4c). To examine, whether Wnt signatures differ also between human colon cancer samples from different locations, we compared again MSS tumours with a mutation in *APC* from TCGA data. Among the Wnt target genes which show a trend to differential expression between left- and right-sided colon cancer, we found most genes to be upregulated more in left-sided cancer samples, however a few genes were more activated in the right-sided colon cancer samples (Additional file 4C). This is in line with previous reports that Wnt activation is higher in distal colon cancer [52, 53]. It also gives the indication that Wnt response is qualitatively location dependent even in patients samples.

## Discussion

In this study we set out to disentangle the impact of the cell that transforms, and the effects of the pathway that drives carcinogenesis on the resulting gene expression profile. For this fundamental research question, we employed a model system with low complexity in order to reduce the confounding factors. The organoid model allowed us to study the epithelial component of early carcinogenesis through titrated Wnt activation. To achieve this, we transduced the stabilized version of *Ctnnb1* downstream of APC, because *Apc* requires biallelic mutations for cancer initiating Wnt activation which seem to (inter)depend on the intestinal location [54]. With organoids from three different murine intestinal locations we defined regional identity markers using RNA sequencing data, extending earlier studies of others [20]. These location identity markers were concealed after Wnt activation via *Ctnnb1*^*S*^ as compared to the original wild type organoids. As expected, cell type differentiation markers were downregulated [55]. The upregulated Wnt target genes differed between the locations and were generally lowly expressed in the control organoids. It was previously reported that the intestinal axis from proximal to distal needs an increasing dose of added Wnt signal to exceed the polyp initiation threshold [21], which was in our model reflected by an increasing amount of mutated *Ctnnb1*^*S*^. The lower levels of *Ctnnb1*^*S*^ in prSI might explain why results were less pronounced, yet significant, in this location. To validate the importance of our findings for the disease we were modelling, we examined human normal colon and colon carcinoma data from distinct colon regions. Because variation between left and right sided colon cancer is tightly connected to differences in pathomechanisms (reviewed in [22]), we narrowed down the problem by focussing on MSS Wnt activated colon cancer samples to unveil the role of region-specific transcriptional background. When we compared normal and cancer samples from the left and right colon, we could recapitulate both of our main observations from the murine organoid model: concealing of location markers and location differences in known Wnt target genes.

Our analyses reveal that there is a profound impact of the region-specific cellular features on the transcriptomic profiles of the resulting tumours, at least along the different locations of the intestinal tract. However, unlike previously assumed, cells do not directly carry over their cell specific gene expression profiles, but reveal initially hidden region-specific signatures. This has implications for the concept of the cell of origin in cancer as well as for the methods used to study this phenomenon. Our data suggest that cell or tissue specific profiles from non-transformed tissues are non-informative to predict the region of origin of a given cancer. Our results indicate that the region-specific features of cells can be partially erased, while the region of origin does have an important impact on defining which genes are activated upon oncogenic pathway activity.

Several limitations of this study include the fact that we have only evaluated the effect of a Wnt activating mutation in specific regions of the intestine. It remains to be defined if our results can be generalized to other oncogenic pathways and organs. For example, *KRAS* mutations are more prevalent in the proximal colon and it has been recently suggested that certain *KRAS* mutations might be location dependent [56, 57]. Moreover, *RAS* mutations have a synergistic effect with *APC* mutations [58] so that the further examination of location dependencies in other oncogenic pathways should incorporate potential cooperative effects. Another limitation is that we only studied the impact of the region of origin on the longitudinal axis in the intestine. It remains unclear how the profiles of distinct cell types along the differentiation axis in the intestine, e.g. Lgr5^+^ stem cells, absorptive progenitors or neuroendocrine cells, shape the phenotype of the resulting neoplasm. This is an important unresolved question, in particular because an increasing range of cell types is identified as amendable to transformation, usually as a result of inflammation associated signals from the environment [59, 60]. It remains an attractive explanation for the notion that the highly diverse phenotypes of colon cancers can only partially be explained by genetic differences. On the other hand, the cell type specific differentiation gradient is already known to be convertible, involving major changes in chromatin accessibility but maintaining a widely stable epigenetic status [61–63]. In that way, it seems less likely that differentiation-related features are hardwired and conserved in cancers of the intestine.

To conclude, at a very basic level, our results provide further support to the fact that distinct regions in the gut present with distinct features. Some of these are potentially clinically significant. For example, tumour location in the colon is associated with response to anti-EGFR agents in the RAS/RAF wild type population. Whilst left-sided cancers display marked clinical benefit, right-sided tumours are less responsive [64, 65]. These observations might be associated with regional differences that are exposed following oncogenic pathway activation. Hence, our findings provide a way forward in an attempt to resolve this notion.

## Conclusion

We observed that region-related cellular identity has a substantial effect on the reaction to Wnt activation in a simple intestinal adenoma model. While the tissue specific pattern is partially erased, the response to oncogenic transformation cannot be inferred from the tissue of origin. These findings highlight the context dependent effect of oncogenic Wnt signalling and provide a way forward in resolving the distinct biology between left- and right-sided human colon cancer with potential clinical relevance.

## Methods

### Organoid generation and maintenance

Isolation of small intestine (SI) and colon crypts from wild type mice was adapted from Sato et al. [66]. Briefly, resected prSI, diSI and colon were opened longitudinally, villi were removed and cut into 0.5 mm pieces. The fragments were washed several times with cold PBS. After this, the intestinal pieces were incubated in 2 mM (SI) or 25 mM (colon) EDTA in PBS at 4 °C for 30 min, in order to detach intestinal crypts from the basal layer. After removal of EDTA, crypts were resuspended in 10 mL of cold PBS 10% FCS, pipetted up and down vigorously, and passed through a 70 μm strainer. Subsequently, crypts were resuspended in Matrigel® (Corning), seeded in 24-well plates, and supplemented with Advanced DMEM/F12 medium (Gibco) containing 1× N2 (Gibco), 1× B27 (Gibco) 50 ng/mL, 10 mM HEPES (Boehringer), Glutamax (Gibco) and 1 mM N-acetylcysteine (Sigma-Aldrich), supplemented with 50 ng/mL murine EGF (TEBU-Bio) and 10% of Noggin and R-spondin1 conditioned media (hereafter called ENR medium). In addition, 10 μM CHIR (Axon Medchem) and 10 μM Rock inhibitor (Sigma-Aldrich) were added to all crypt cultures. After two days in culture, CHIR was removed from SI crypt cultures and Rock inhibitor from both, SI and colon. Cultures were incubated at 37 °C and 5% CO_2_.

### Generation of Ctnnb1^S^ construct

A stabilized (mutated to p.S33A;S37A;T41A;S45A) version of *Ctnnb1* (GenBank ID: NM_001165902.1:c.[97T > G;109T > G;121A > G;13T > G]) was obtained in pLX304 as a gift from William Hahn (Addgene # 42561) and cloned into the vector pWPXL downstream the constitutively active promotor EF1α (Addgene #12,257) which was kindly provided by Didier Trono Finally, P2A-dsRed sequence was cloned downstream *Ctnnb1*^*S*^ gene. All restriction enzymes were from New England Biolab. Lentiviral particles were generated using PEI transfection with 2nd generation packaging plasmids VSV-G pMD2.G (Addgene #12259) and PAX2 (Addgene #12260), kindly provided by Didier Trono respectively.

### Generation of adenoma organoids

Wild type organoids from prSI, diSI and colon were transduced with the pWPXL-Ctnnb1^S^-P2A-dsRed plasmid in order to generate adenoma organoids [67, 68]. Once adenoma cultures were established, selection for positively transduced organoids was performed. Since the Wnt pathway is upregulated in these organoids, the dependence on external Wnt agonist is diminished. Therefore, after four days selection for pWPXL-Ctnnb1^S^-P2A-dsRed positive organoids was performed by culturing them in selection medium, ENR without R-spondin1 conditioned medium for SI and ENR for colon organoids. Visual expression of the dsRed construct by fluorescence microscopy (Zeiss AxioVert 200) confirmed successful transduction (Additional file 1A). We further confirmed *Ctnnb1* upregulation in quantitative PCR, Western Blot and on RNA sequencing data.

### Quantitative PCR

Overexpression of *Ctnnb1*, detection of Wnt targets and specific region markers were validated by quantitative PCR. In short, RNA was isolated using NucleoSpin® RNA kit (Macherey–Nagel). Then, 500 ng of total RNA were retrotranscribed using 5 μg/mL polydT primers and SuperScript® III reverse transcriptase (Thermo Scientific) at 50 °C. cDNA was diluted 1:20 in distilled water and amplified in 5 μL reactions with LightCycler® 480 SYBR Green I Master 2X on a LightCycler® 480 (Roche). Results were analysed using LightCycler® 480 software version 1.5.1.62. Relative fold gene expression was calculated using the delta-delta Ct (2^−DDCt^) method [69]. Primer sequences can be found in Additional file 5.

### Western blotting

Organoid protein lysates were made using Cell Lysis Buffer (Cell Signaling Technologies) according to manufacturers’ protocol. Subsequently, protein concentrations were determined using Pierce^™^ Protein Assay Kit (Thermo Scientific). Next, 30 μg protein was loaded in 4–15% Mini-PROTEAN® TGX precast protein gels (Bio-Rad), separated by electrophoresis and transferred to PDVF membranes using the Trans-Blot Turbo System (Bio-Rad). Membranes were blocked in 5% Skim Milk Powder (Sigma) and primary antibodies were incubated overnight in 5% BSA/TBST and washed with TBST (0.1% Tween® 20, P1379, Sigma). HRP-conjugated secondary antibodies were incubated for 1 h at room temperature in 5% BSA/TBST and washed with TBST. Protein levels were detected using Pierce^™^ ECL Western Blotting Substrate (Thermo Scientific) and revealed using ImageQuant LAS 4000 (GE Healthcare Life Sciences). Primary antibodies used are anti-β-Catenin (9562, Cell Signaling Technologies), anti-REG1A (PA5-70,626, Thermo Scientific), anti-FABP6 (PA5-50,407, Thermo Scientific), anti-MUC1 (PA5-95,487, Thermo Scientific) and anti-GAPDH (MAB374, Millipore). Secondary antibodies are anti-rabbit-HRP (7074, Cell Signaling technologies) and anti-mouse-HRP (1070–05, Southern Biotech). Images were cropped around the target protein sizes. Loading control was performed with GAPDH staining for all membranes.

### RNA sequencing

Total RNA was isolated using NucleoSpin RNA isolation kit (BIOKÉ, Leiden, Netherlands) and the quality of RNA samples was subsequently assessed using Agilent 2100 Bioanalyzer (Agilent Technologies Inc., Santa Clara, CA, USA). Generation of sequencing libraries was performed using the TruSeq RNA Library Preparation Kit v2 (Illumina, San Diego, CA, USA) and 1 μg of total RNA. Sequencing was performed on HiSeq2500 (Illumina) with 50-bp single-end reads. Average sequencing depth (total number of reads) was 17.69 M reads (range 11.28 M to 23.32 M). Mean coverage (number of reads × read length / target size) of all genes was on average 4.395 (range 2.658 to 6.480). After excluding genes with a mean read count < 1 for downstream analysis, the average coverage across the samples was 11.293 (range 6.831 to 16.654).

### Data retrieval

Reads were aligned and quantified using HISAT2 (2.0.4), samtools (1.3.1) and subread featureCounts (1.5.0-p1) with GRCm38.85 [70–72]. The mutated region of *Ctnnb1*^*S*^ comprising four mutations was identified among the unmapped reads as a 37b long string “*G*CTGGAATCCAT*G*CTGGTGCCACC*G*CCACAGCTCCT*G*” as opposed to the wild type “TCTGGAATCCATTCTGGTGCCACCACCACAGCTCCTT” among the mapped reads.

TCGA gene expression data as raw counts and clinical data was obtained using TCGAbiolinks (2.12.3) [73]. Only samples with an annotated tumour site or site of resection or biopsy were analysed, assigning sigmoid and descending colon to left colon and cecum and ascending colon to right colon. MSS tumours with a somatic *APC* mutation were selected based on the annotations of the original publication [8]. CMS labels were obtained from the original publication [16].

### Data analysis

DESeq2 (1.10.1) was applied for normalization and differential expression analysis [74]. The results were annotated with org.Mm.eg.db (3.8.2) or org.Hs.eg.db (3.8.2) and plotted using ComplexHeatmap (2.0.0) or ggplot2 (3.2.0) [75]. After ranking genes according to the decreasing Wald statistics value, GseaPreranked (v3.0) was used for gene set enrichment analysis on ENCODE transcription factor target gene sets, Wnt target genes and location markers from literature as well as gene lists generated from our own data using cut-offs of fold change > 1.2 and Benjamini–Hochberg adjusted p-value of 0.05 [20, 32, 33, 51, 74]. Results were replotted adapting Rtoolbox::replotGSEA (v.1.4). An alternative Gene Set Enrichment method based gene expression analysis with limma::voom(3.40.2) and edgeR (3.26.5) was used on MiSigDB Hallmark gene sets with EGSEA (1.12.0) [49, 50, 76–78]. Overlapping transcription factor target gene sets were identified using ChEA3 mean rank mode on the 200 genes with the highest significant upregulation (fold change > 2, adjusted p-value < 0.05) between Ctnnb1^S^ and wt in each location or between the left and right normal colon from TCGA samples [74, 79]. The score was inverted in order to be maximal with the highest overlap evidence. From genes coding these transcription factor we selected genes that showed variance in expression in wild type and in the log2 fold change upon Wnt activation between the locations (above average) and plotted their correlation to the corresponding transcription factor targets overlap. Correlation was tested using the Spearman method in R package ggpubr (0.2.5).

## Supplementary information

**Additional file 1.** Validation of findings. (A) The expression of dsRed after Ctnnb1^S^ transduction was examined by fluorescence microscopy including images merged with bright field, scale bar = 200 μm. (B) Heatmap depicts average (n = 3) scaled counts for both conditions of each location, right columns show summarized log2 fold changes, log2FC(Cβ/wt), and mean read count across locations. Genes represent our location signatures comprising each 200 most significantly (Wald-Test) upregulated genes comparing the wt samples of prSI, diSI or colon to all other locations’ wt samples, respectively. (C) Validation of location marker genes with quantitative PCR, n = 3, Student’s t-test, error bars represent S.E.M., mean fold change in vertical number. (D) Immunoblot of prSI location marker REG1, diSI location marker FABP6, colon location marker MUC1 and control GAPDH, protein lysates from organoids grown without (wt) or with transduced Ctnnb1^S^ (Cβ). The loading control is exemplarily shown for the FABP6 membrane. (E) For genes which are significantly differentially expressed in the murine organoid locations in colon vs. small intestine and in TCGA normal samples from the left vs. right colon we find a significant correlation (Spearman) of the log2 fold changes (log2FC).

**Additional file 2.** Location signatures are downregulated upon Wnt activation. Genes depicted in Fig. 2A are provided in a table format with compiled log2 fold changes (l2FC) (n = 3) for each location separately. The comparison of the wt samples of prSI, diSI or colon to all other locations’ wt samples retrieved the most significantly (Wald-Test) upregulated genes categorized as internal location (int. loc.) marker genes. Corresponding log2 fold changes are listed under l2FC.loc for all locations. External location (ext. loc.) marker genes were previously reported to be specific to intestinal divisions [20, 33] or specifically enriched in the intestine based on the Human Protein Atlas [34–37]. Cell type marker genes were previously reported to be specific to differentiated intestinal cells [38–40]. The downregulation of location and differentiation markers upon Wnt activation can be appreciated in the separately calculated log2 fold changes (Cβ/wt) listed under l2FC.ctn.vs.wt. NA values correspond to expression below detection in either condition or a non-mappable gene symbol from literature.

**Additional file 3.** Location signatures are downregulated upon Wnt activation in vivo. (A) To validate our findings in human in vivo samples we used TCGA data [8] with the summarized clinical characteristics of patients. *Transverse colon samples were depicted but not included in the differential expression analyses between the two sides, due to lack of more specific site information. The same holds for a single sample from normal transverse colon of a female patient (44 years). (B) Using transcriptome data from TCGA [8] we identified genes differentially expressed (adjusted p-value < 0.01, Wald-Test) between left and right colon in the normal tissue samples (left panel). The expression of those genes in MSS *APC* mutated colon adenocarcinoma samples is shown in the right panel. Samples for transverse colon are included for comparison purpose. (C) Transcription factors for which an enrichment of their target genes was observed in normal colon side-specific genes show little or no differential expression of their own coding genes in the normal colon but partially a differential expression between normal colon and colon cancer samples.

**Additional file 4.** Wnt response is partly location specific. (A) Heatmap with average (n = 3) scaled counts, centred around the location mean. Right columns show summarized log2FC(Cβ/wt) and mean read count across locations. Using a likelihood ratio test, we identified genes, in which the reaction to Wnt activation depends significantly on the location. The location dependently upregulated genes were separated into 3 groups, where the upregulation was maximal in prSI, diSI or colon, respectively. (B) Genes differentially expressed between left and right TCGA colon cancer samples were tested for enrichment in Hallmark genes sets using EGSEA. (C) Using transcriptome data from TCGA [8] we tested, which Wnt target genes [32] were differentially expressed between left and right colon in the MSS *APC* mutated colon cancer samples. Wnt genes with a trend to differential expression between the colon cancer locations (adjusted p-value < 0.05 at *TP53RK*, all genes p-value < 0.15, Wald-Test) are plotted (right panel). The expression of those genes in normal samples is shown in the left panel. Samples for transverse colon are included for comparison purpose.

**Additional file 5.** Primers used in quantitative PCR.

## Data Availability

The dataset generated and analysed during the current study is available in the Gene Expression Omnibus (GEO) repository, under accession number GSE146476 (https://www.ncbi.nlm.nih.gov/geo/query/acc.cgi?acc=GSE146476).

## Abbreviations

CMS: Consensus molecular subtype
diSI: Distal small intestine
GSEA: Gene set enrichment analysis
MSS: Microsatellite stable
prSI: Proximal small intestine
SI: Small intestine
Cβ, Ctnnb1^S^: Stabilized β-catenin
TCGA: The Cancer Genome Atlas
wt: Wild type
