# Supplementary figures and images for: Intestinal region-specific Wnt signalling profiles reveal interrelation between cell identity and oncogenic pathway activity in cancer development
Source: Cancer Cell Int. 2020 Dec 3;20:578. doi: 10.1186/s12935-020-01661-6 (PMC7713000; doi:10.1186/s12935-020-01661-6)

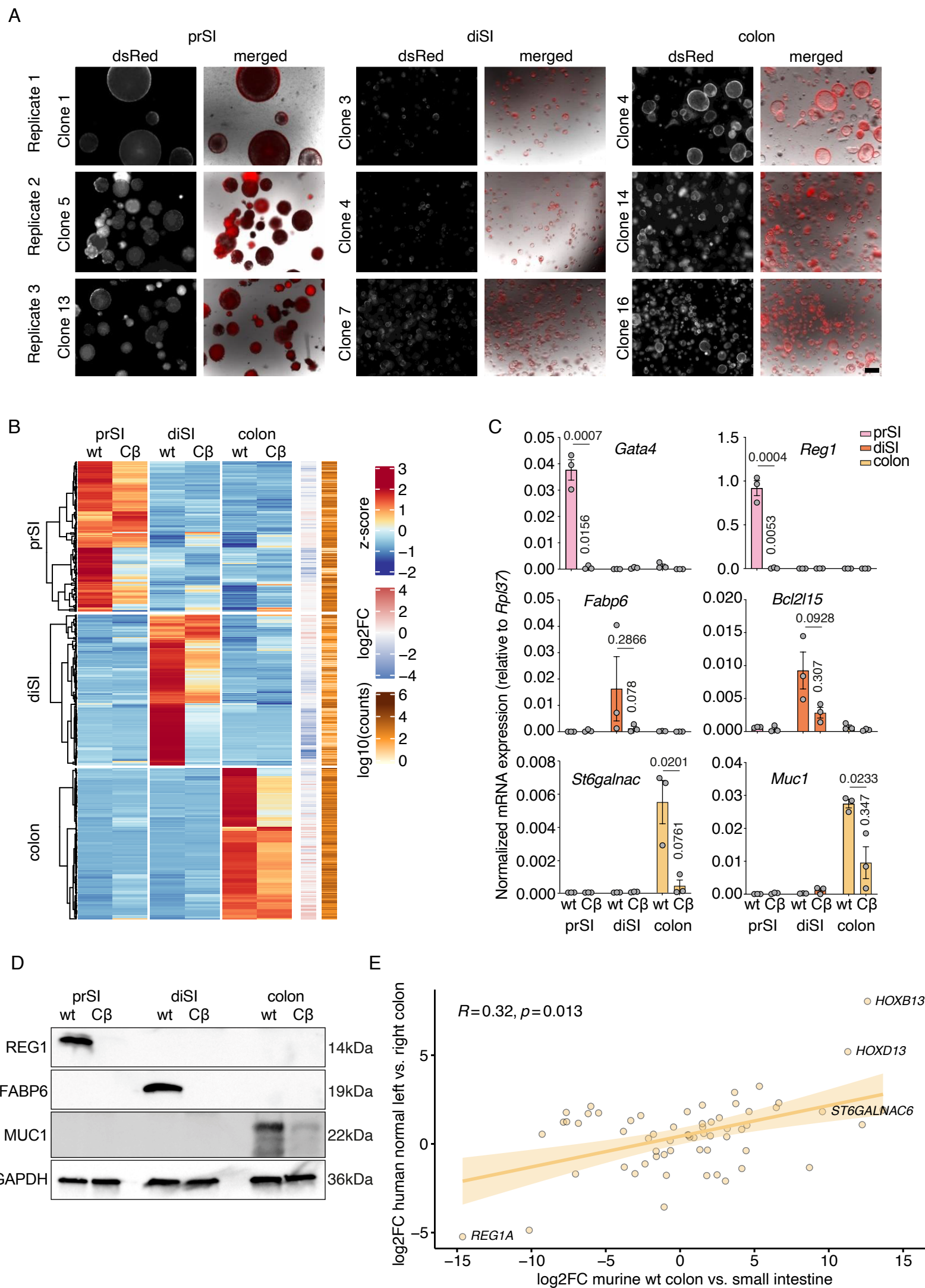

Supplement: Supplementary file 1 — Additional file 1. Validation of findings. (A) The expression of dsRed after Ctnnb1S transduction was examined by fluorescence microscopy including images merged with bright field, scale bar = 200 μm. (B) Heatmap depicts average (n = 3) scaled counts for both conditions of each location, right columns show summarized log2 fold changes, log2FC(Cβ/wt), and mean read count across locations. Genes represent our location signatures comprising each 200 most significantly (Wald-Test) upregulated genes comparing the wt samples of prSI, diSI or colon to all other locations’ wt samples, respectively. (C) Validation of location marker genes with quantitative PCR, n = 3, Student’s t-test, error bars represent S.E.M., mean fold change in vertical number. (D) Immunoblot of prSI location marker REG1, diSI location marker FABP6, colon location marker MUC1 and control GAPDH, protein lysates from organoids grown without (wt) or with transduced Ctnnb1S (Cβ). The loading control is exemplarily shown for the FABP6 membrane. (E) For genes which are significantly differentially expressed in the murine organoid locations in colon vs. small intestine and in TCGA normal samples from the left vs. right colon we find a significant correlation (Spearman) of the log2 fold changes (log2FC). [file 12935_2020_1661_MOESM1_ESM.pdf]

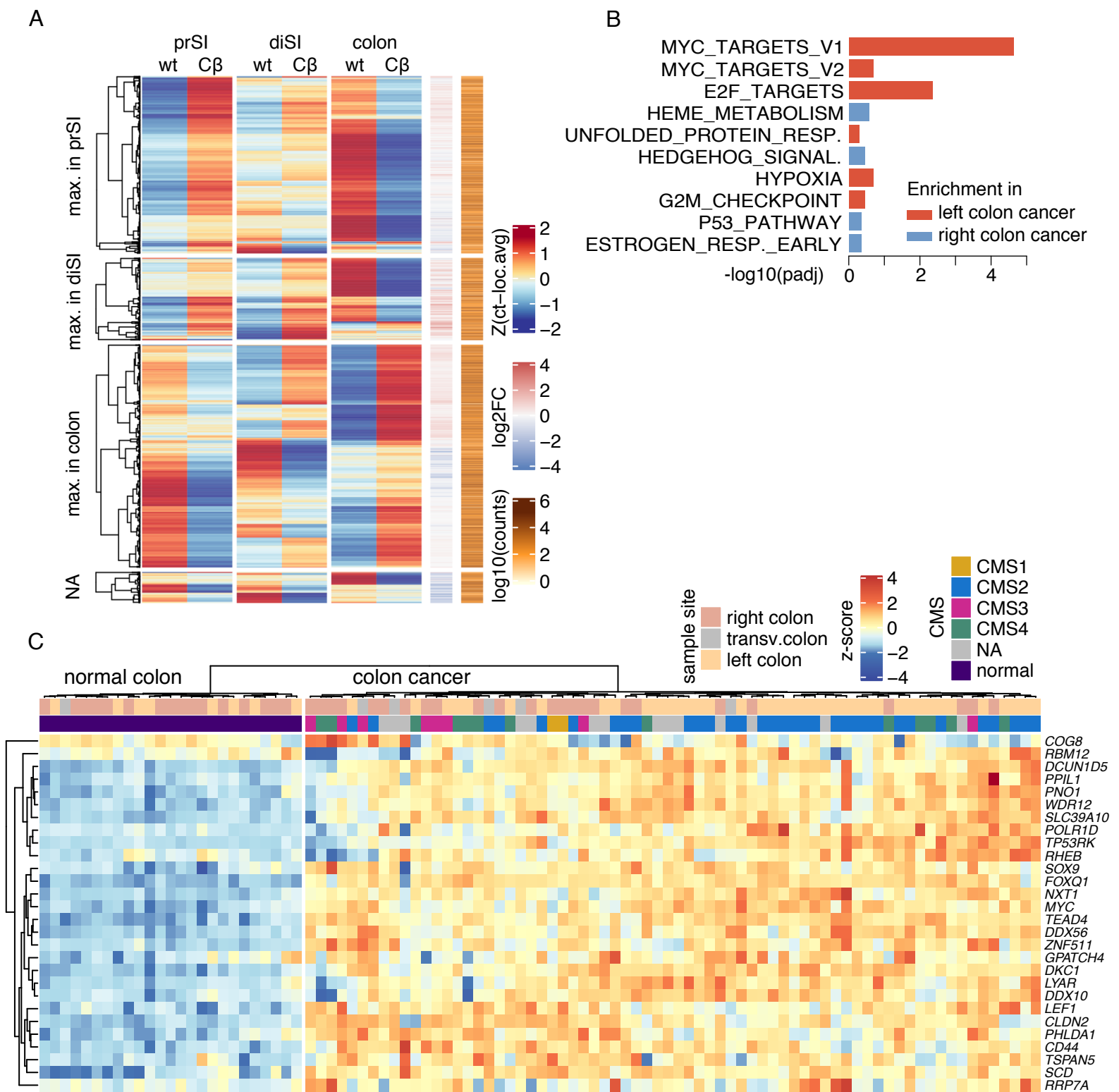

Supplement: Supplementary file 4 — Additional file 4. Wnt response is partly location specific. (A) Heatmap with average (n = 3) scaled counts, centred around the location mean. Right columns show summarized log2FC(Cβ/wt) and mean read count across locations. Using a likelihood ratio test, we identified genes, in which the reaction to Wnt activation depends significantly on the location. The location dependently upregulated genes were separated into 3 groups, where the upregulation was maximal in prSI, diSI or colon, respectively. (B) Genes differentially expressed between left and right TCGA colon cancer samples were tested for enrichment in Hallmark genes sets using EGSEA. (C) Using transcriptome data from TCGA [8] we tested, which Wnt target genes [32] were differentially expressed between left and right colon in the MSS APC mutated colon cancer samples. Wnt genes with a trend to differential expression between the colon cancer locations (adjusted p-value < 0.05 at TP53RK, all genes p-value < 0.15, Wald-Test) are plotted (right panel). The expression of those genes in normal samples is shown in the left panel. Samples for transverse colon are included for comparison purpose. [file 12935_2020_1661_MOESM4_ESM.pdf]
