# Supplementary material for: Intestinal region-specific Wnt signalling profiles reveal interrelation between cell identity and oncogenic pathway activity in cancer development
Source: Cancer Cell Int. 2020 Dec 3;20:578. doi: 10.1186/s12935-020-01661-6 (PMC7713000; doi:10.1186/s12935-020-01661-6)
Supplement: Supplementary file 3 — Additional file 3. Location signatures are downregulated upon Wnt activation in vivo. (A) To validate our findings in human in vivo samples we used TCGA data [8] with the summarized clinical characteristics of patients. *Transverse colon samples were depicted but not included in the differential expression analyses between the two sides, due to lack of more specific site information. The same holds for a single sample from normal transverse colon of a female patient (44 years). (B) Using transcriptome data from TCGA [8] we identified genes differentially expressed (adjusted p-value < 0.01, Wald-Test) between left and right colon in the normal tissue samples (left panel). The expression of those genes in MSS APC mutated colon adenocarcinoma samples is shown in the right panel. Samples for transverse colon are included for comparison purpose. (C) Transcription factors for which an enrichment of their target genes was observed in normal colon side-specific genes show little or no differential expression of their own coding genes in the normal colon but partially a differential expression between normal colon and colon cancer samples. [file 12935_2020_1661_MOESM3_ESM.pdf]

A

Characteristic		normal colon		colon cancer		
		Right (n=16)	Left (n=8)	Right (n=25)	Transv. (n=5)*	Left (n=40)
**Gender**	Female (%)	56.25	25.0	44	20	42.5
	Male (%)	43.75	75.0	56	80	57.5
**Age**	Median (years)	78	69	71	69	70
	Mean (min-max)	76 (43-90)	68 (40-86)	73 (50-90)	71 (84-53)	69 (38-89)
**Stage**	Stage I (%)			16	20	20
	Stage II (%)			40	60	30
	Stage III (%)			28	20	30
	Stage IV (%)			16	0	20
**Genetics**	APC Mut. (%)			100	100	100
	MSS (%)			100	100	100
	RAS/BRAF Mut. (%)			80	60	30
**CMS**	CMS1 (%)			8	0	0
	CMS2 (%)			24	40	62.5
	CMS3 (%)			24	20	2.5
	CMS4 (%)			16	40	22.5

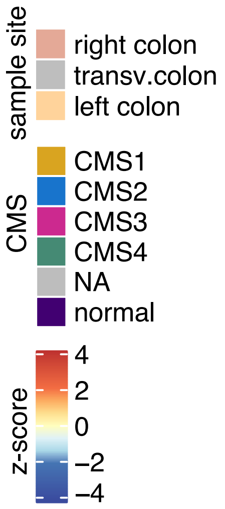

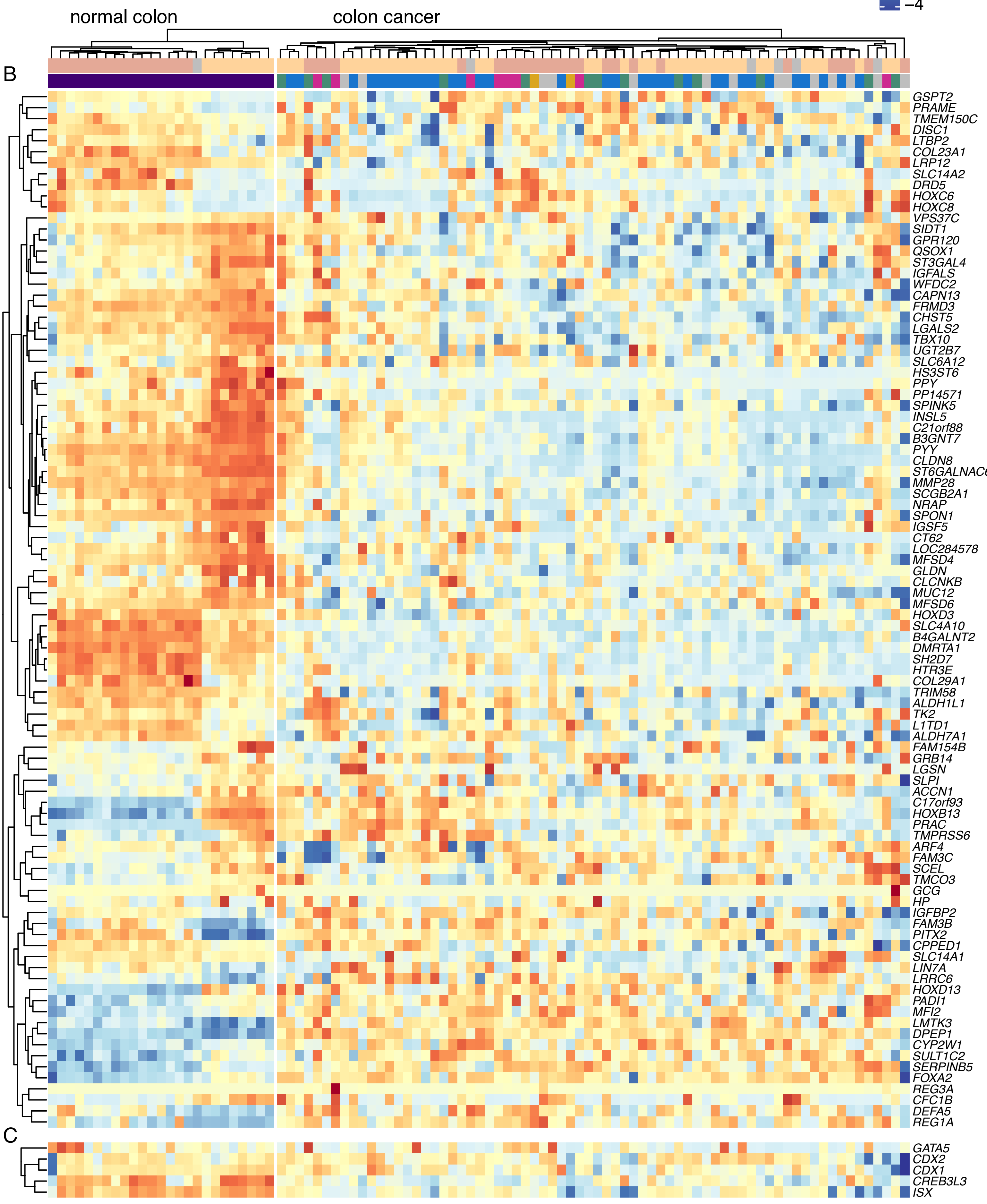

Additional file 3
